# Long non-coding RNAs in bone formation: Key regulators and therapeutic prospects

**DOI:** 10.1515/biol-2022-0908

**Published:** 2024-08-16

**Authors:** Chun Jiang, Peng Wang, ZhenWei Tan, Yin Zhang

**Affiliations:** Department of Orthopedics, The People’s Hospital of SND, Suzhou, Jiangsu, 215129, China; Department of Spine Surgery, Shengli Oilfield Central Hospital, Dongying, Shandong, 257000, China; Department of Orthopedics, Sichuan Fifth People’s Hospital, Chengdu, Sichuan, 610015, China

**Keywords:** long non-coding RNAs, bone formation, osteoblast differentiation, Runx2, osteoporosis

## Abstract

Recent scientific investigations have revealed the intricate mechanisms underlying bone formation, emphasizing the essential role of long non-coding RNAs (lncRNAs) as critical regulators. This process, essential for skeletal strength and functionality, involves the transformation of mesenchymal stem cells into osteoblasts and subsequent deposition of bone matrix. lncRNAs, including HOX transcript antisense RNA (HOTAIR), metastasis-associated lung adenocarcinoma transcript 1 (MALAT1), differentiation antagonizing non-coding RNA (DANCR), and maternally expressed gene 3 (MEG3), have emerged as prominent players in this regulatory network. HOTAIR modulates osteoblast differentiation by interacting with chromatin-modifying enzymes, while MALAT1 regulates osteogenic differentiation through microRNA interactions. DANCR collaborates with Runx2 to fine-tune osteoblast differentiation, and MEG3 orchestrates multiple signaling pathways crucial for bone formation. Moreover, other lncRNAs such as H19, lncRNA for enhancing osteogenesis 3, rhabdomyosarcoma 2-associated transcript, urothelial cancer associated 1, taurine up-regulated gene 1, and nuclear enriched abundant transcript 1 contribute to the complex regulatory network governing osteoblast activities. Understanding the precise roles of these lncRNAs offers promising avenues for developing innovative therapeutic strategies targeting bone-related disorders like osteoporosis. Overall, this review summarizes the pivotal role of lncRNAs in bone formation, highlighting their potential as targets for future research endeavors aimed at advancing therapeutic interventions in bone diseases.

## 1 Introduction

The process of bone formation requires the precise coordination of osteoblast differentiation, matrix mineralization, and bone remodeling. Essential factors in this process include Runx2 and Osterix, which are well-recognized for their roles in osteoblast differentiation and bone formation. Recent studies have identified various long non-coding RNAs (lncRNAs) that serve as regulators for these transcription factors and other genes involved in bone formation [1,2]. lncRNAs, defined as RNAs exceeding 200 nucleotides in length but lacking protein-coding capacity, were initially disregarded as mere transcriptional noise generated during RNA polymerase II transcription. However, accumulating evidence now highlights the crucial functions of lncRNAs as integral regulators of nuclear chromatin structure and gene expression, both in the context of development and during various pathological conditions.

In the past, lncRNAs were often dismissed as inconsequential transcriptional byproducts. However, contemporary research has unveiled the substantial roles that lncRNAs play in a wide spectrum of cellular functions. These functions encompass pivotal activities such as genomic imprinting, manipulation of chromosomal structure, and the allosteric modulation of enzyme behavior [3]. The influence of lncRNA expression extends to a diverse array of cellular processes, including the regulation of the cell cycle, cell proliferation, metastasis, immunobiological responses, and cellular differentiation [4]. lncRNAs employ a versatile array of mechanisms, functioning as scaffolds, decoys, signals, and guides. These fundamental prototypes represent the molecular underpinnings of lncRNA activities, which may operate independently or in combination. Currently, lncRNAs are predominantly recognized for their roles in regulating cellular processes through RNA-protein, RNA-RNA, and RNA-DNA interactions [5]. In addition to facilitating the precise localization of chromatin regulatory proteins, lncRNAs also orchestrate the formation of RNA-protein complexes, thereby recruiting functional proteins possessing diverse protein interaction domains [6]. lncRNAs are known to modulate an assortment of processes, including pre-mRNA splicing, RNA editing, regulation of mRNA stability, activation of translation, and acting as sponges for miRNAs [7]. Furthermore, lncRNAs exert regulatory control over biological processes by selectively targeting specific DNA sequences through the formation of RNA–DNA triplexes. A mounting body of research [8] underscores the pivotal roles played by lncRNAs in the onset and progression of various human disorders.

Although most lncRNAs are produced only at specific times during the life cycle of a given cell or tissue, their partial transcripts are widely and constitutively expressed. lncRNAs are produced at lower levels than mRNAs and can be found in both the nucleus and cytoplasm of a cell. However, lncRNAs do exhibit some degree of conservation in their promoter and exon regions, suggesting that their functions are relatively conserved, unlike miRNAs, which display much higher interspecies homology. In contrast to the 1% of the human genome that codes for proteins, lncRNAs constitute 49% of the genome’s sequence. The biogenesis and mechanisms of lncRNAs have been extensively studied in previous literature [7,9]. This review focuses on exploring the potential of specific lncRNAs as targets for promoting osteogenic differentiation and bone regeneration.

## 2 HOX transcript antisense RNA (HOTAIR)

On chromosome 12, HOXC transcribes HOTAIR. In breast cancer, it recruits chromatin-modifying enzymes to gene loci. HOTAIR is now known to be involved in stem cell differentiation, epigenetic control, and immune system function. By utilizing chromatin-modifying enzymes, this lncRNA regulates osteoblast differentiation and bone formation. Several studies have shown that HOTAIR controls osteoblast differentiation and the accumulation of bone matrix during bone formation. The expression of HOTAIR has been reported to be elevated in various cancers, and its overexpression is associated with tumor growth, invasion, and metastasis. It has been discovered that HOTAIR regulates the expression of important transcription factors and signaling pathways related to osteoblast development. Additionally, HOTAIR has the ability to function as a molecular scaffold, aiding in the formation of protein complexes necessary for osteoblast development. Gaining an understanding of the molecular aspects of HOTAIR’s role in this process could lead to the development of innovative treatment approaches for conditions connected to bone. However, recent studies have also revealed HOTAIR’s involvement in other diseases, such as osteonecrosis and developmental disorders.

Li et al. demonstrated that targeted disruption of Hotair in mice led to homeotic transformation and gene derepression [10]. The researchers found that Hotair knockout mice exhibited developmental defects, including a shift in the expression of Hox genes, which are critical regulators of developmental processes. The study provided evidence that HOTAIR plays a crucial role in regulating gene expression during development. In addition to its role in development, HOTAIR has been implicated in the pathogenesis of osteonecrosis, a condition characterized by the death of bone tissue due to a lack of blood supply. In the case of non-traumatic osteonecrosis of the femoral head, Wei et al. found that HOTAIR suppresses miR-17-5p to control osteogenic differentiation and proliferation [11]. The researchers observed that HOTAIR expression was upregulated in osteonecrosis tissues compared to normal tissues. They also noted that HOTAIR knockdown promoted osteogenic differentiation and inhibited cell proliferation in osteonecrosis-derived bone marrow mesenchymal stem cells (BMSCs). These findings suggest that HOTAIR plays a role in the pathogenesis of osteonecrosis by regulating osteogenic differentiation and proliferation. Shen et al. investigated the role of HOTAIR in the osteogenic differentiation of BMSCs [12]. By modulating the Wnt/beta-catenin pathway, the researchers found that HOTAIR suppressed BMSCs’ ability to differentiate into osteoblasts. They also observed that HOTAIR knockdown promoted osteogenic differentiation of BMSCs, while HOTAIR overexpression had the opposite effect. These findings suggest that HOTAIR plays a negative role in the osteogenic differentiation of BMSCs and may contribute to the development of osteoporosis and other bone-related diseases.

In conclusion, HOTAIR is an lncRNA involved in various biological processes, including development, gene expression, and disease pathogenesis. The studies reviewed here suggest that HOTAIR plays a role in the development of various diseases, including cancer, osteonecrosis, and osteoporosis. While much remains to be learned about the precise mechanisms underlying HOTAIR’s functions, these findings highlight the molecule’s importance in regulating cellular processes and disease pathogenesis. Osteoblast differentiation and bone formation are impacted by HOTAIR’s essential function in epigenetically controlling osteogenic gene expression. Knowing its mechanisms can help develop new treatment strategies to address bone problems. These strategies may allow for targeted interventions to modify the processes that lead to bone remodeling and treat conditions such as osteoporosis and fractures. Further research on HOTAIR and its functions may lead to the development of novel therapeutic strategies for a wide range of diseases.

## 3 Metastasis-associated lung adenocarcinoma transcript 1 (MALAT1)

The lncRNA MALAT1 was initially associated with predicting lung cancer metastasis. Since then, MALAT1 has been implicated in numerous cellular processes, including cell growth, migration, differentiation, and diseases such as cancer, cardiovascular diseases, and neurodegenerative conditions. Recent research has also linked MALAT1 to bone growth and suggested its involvement in osteogenic differentiation, potentially contributing to the development of osteoporosis. MALAT1 controls chromatin architecture, signaling pathway modulation, gene expression regulation, and osteoblast maturation during osteogenic differentiation. Recognizing its function helps to clarify the processes controlling bone homeostasis and skeletal development.

Zhou et al. delved into MALAT1’s role in osteogenic differentiation in osteoporosis [13]. Their study revealed that MALAT1 mediates osteogenic differentiation by regulating the miR-485-5p/WNT7B axis. MALAT1 expression was found to be elevated in osteoporotic bone tissues, and its knockdown inhibited the osteogenic differentiation of BMSCs. Furthermore, MALAT1 knockdown reduced the expression of WNT7B, a key osteogenic regulator, through miR-485-5p mediation. These findings suggest MALAT1’s involvement in osteoporosis pathogenesis via the miR-485-5p/WNT7B axis. In human BMSCs, MALAT1 was shown to upregulate osterix expression, thereby positively influencing osteogenic differentiation [14]. During BMSC osteogenic differentiation, MALAT1 expression increased, and its knockdown hindered this process. Additionally, MALAT1 regulated osterix expression by targeting miRNA-143, which acts as a negative regulator of osterix. This evidence suggests that MALAT1 plays a constructive role in osteogenic differentiation by modulating osterix expression.

Zhang et al. explored the function of MALAT1 in regulating osteo-lineage differentiation of BMSCs by investigating its interaction with microRNA-124 (miR-124) [15]. Their study revealed that MALAT1 binds to miR-124 and modulates the expression of Runx2, a pivotal regulator of osteogenic differentiation. MALAT1 knockdown was observed to hinder BMSC osteogenic differentiation, with this effect being mediated by miR-124. These findings suggest that MALAT1 contributes to osteogenic differentiation by regulating the interplay between miR-124 and Runx2. Altogether, MALAT1 is an lncRNA implicated in osteogenic differentiation and potentially linked to the pathogenesis of osteoporosis. The studies discussed here propose that MALAT1 governs osteogenic differentiation by targeting key regulators of this process, including WNT7B, osterix, and Runx2. Osteoporosis and bone metastases are two bone disorders that are correlated with MALAT1 dysregulation. Bone homeostasis and osteogenic differentiation are disturbed by its abnormal expression. A possible therapeutic approach to improve bone health, regulate bone remodeling processes, and maybe slow the advancement of bone-related illnesses is to target MALAT1, opening up new treatment options. Further research into the precise mechanisms governing MALAT1’s functions may pave the way for novel therapeutic approaches in managing bone diseases.

## 4 Maternally expressed gene 3 (MEG3)

The article herein presents a comprehensive review of the role played by MEG3 in osteogenic differentiation and bone-related processes. The MEG3 is involved in the differentiation of osteoblasts. It affects osteogenic gene expression and bone production by controlling gene expression, influencing signaling pathways, and interacting with chromatin modifiers. Knowing the function of MEG3 helps us to better understand the molecular processes that control bone homeostasis and skeletal development. To facilitate clearer understanding and enhanced precision, grammatical corrections are hereby proffered. MEG3 contributes to bone formation in addition to other cellular functions. The mother gene responsible for MEG3 regulates cellular growth, mitosis, and apoptosis. Numerous studies indicate that MEG3 plays a critical role in osteogenic differentiation and bone formation. As an lncRNA, MEG3 has been implicated in both osteogenic differentiation and bone diseases. This article examines various studies that have explored the functionalities and mechanisms of MEG3 in bone-related processes. Sun et al. conducted a comprehensive literature review on MEG3, concluding that it regulates the expression of key genes and signaling pathways related to osteogenic differentiation, including BMP signaling, Wnt signaling, and Runx2. The review also revealed that dysregulation of MEG3 is prevalent in a variety of bone diseases such as osteoporosis, osteoarthritis, and bone cancer [16].

Zhao et al. found that MEG3 inhibits osteogenic differentiation of human dental pulp stem cells through regulation of the miR-543/SURF-1/Runx2 axis [17]. Their findings indicated that knocking down MEG3 expression promoted osteogenic differentiation, whereas overexpression of MEG3 yielded the opposite effect. The authors further demonstrated that MEG3 acts as a competing endogenous RNA (ceRNA) by sponging miR-543, thereby regulating SURF-1 and Runx2 expression. Liu et al. demonstrated that downregulation of MEG3 enhanced osteogenic differentiation of BMSCs and facilitated bone repair through activation of the Wnt/β-catenin signaling pathway [18]. Their *in vitro* and *in vivo* experiments showed that MEG3 knockdown increased BMSC proliferation and osteogenic differentiation, whereas its overexpression had the reverse effect. The study posited that MEG3 could serve as a promising therapeutic target for bone regeneration and the treatment of bone-related diseases. In another set of studies by Liu et al., MEG3 was found to regulate osteogenic differentiation in periodontal ligament stem cells (PDLSCs) through the miR-27a-3p/IGF1 axis and in periodontal ligament cells through the Wnt/β-catenin signaling pathway) [19,20].

**Figure 1:**
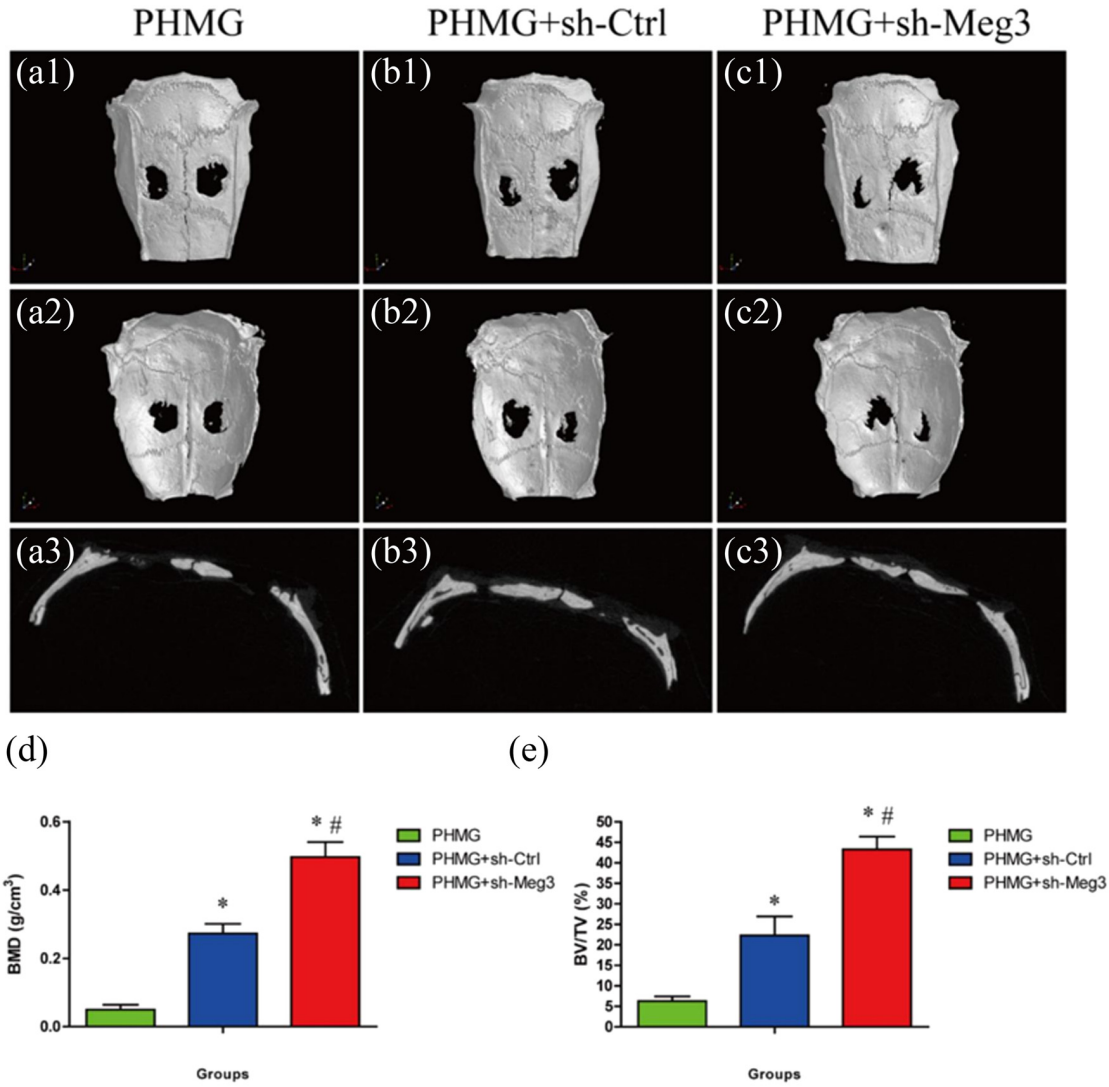
*In vivo* experiments demonstrated that downregulation of MEG3 in BMSCs promoted bone repair. The researchers used micro-CT imaging to compare the bone-repairing ability of PHMG scaffold with BMSCs that had MEG3 knocked down (PHMG + sh-Meg3 group) to control groups (PHMG and PHMG + sh-Ctrl) at 8 weeks. The micro-CT images (a1–c3) showed the representative bone repair in each group. Additionally, (d and e) the bar graph displayed the quantitative analysis of vessel volume measured by micro-CT. Furthermore, the researchers analyzed the bone mineral density and bone volume/total volume (BV/TV) in each group using morphometric analysis, with the results demonstrating significant improvements in the PHMG + sh-Meg3 group compared to the control groups after 8 weeks. Images obtained from Liu et al. [18].

Separately, studies on MALAT1 have indicated its role in promoting osteogenic differentiation. Huang et al. showed that MALAT1 upregulates activating transcription factor 4 by sponging miR-214, thereby enhancing osteogenic differentiation [21]. Yang et al. discovered that MALAT1 elevates the expression of Runx2 through miR-30, promoting osteoblast differentiation [22]. Exosomal MALAT1, as found by Yi et al., enhances osteogenic differentiation through the miR-34c/SATB2 axis [23]. In summation, the reviewed studies underscore the pivotal role that MEG3 plays in regulating osteogenic differentiation and bone pathology. Dysregulation of MEG3 is implicated in various bone diseases, offering a potential therapeutic target for such conditions. Bone disorders like osteoporosis and bone cancers are associated with MEG3 dysregulation. The mechanisms of osteoblast development and bone remodeling are disrupted by aberrant expression. By specifically targeting MEG3, the therapeutic potential is presented for the restoration of normal bone metabolism, the modulation of bone-related signaling pathways, and the development of novel treatments for a variety of bone illnesses, such as osteoporosis and bone cancer. Further research is imperative for a comprehensive understanding of MEG3’s roles in bone biology (Figure 1).

## 5 Differentiation antagonizing non-coding RNA (DANCR)

DANCR is a lncRNA that has been implicated in regulating cell differentiation. DANCR influences the decision of cell destiny during differentiation by controlling gene expression, altering signaling pathways, and interacting with chromatin modifiers. Knowing the function of DANCR helps us understand the mechanisms controlling cellular differentiation in the contexts of development and illness. Its role in the suppression of progenitor differentiation was first described by Kretz et al. [24]. In this study, the researchers found that DANCR is expressed in a tissue-specific manner in progenitor cells, and its overexpression inhibits their differentiation into mature cells. Conversely, the knockdown of DANCR promotes differentiation. The researchers proposed that DANCR acts as a "molecular sponge" for microRNAs involved in the differentiation process, thereby blocking their activity and maintaining the progenitor state.

Subsequent to this initial study, several others have explored the role of DANCR in various differentiation processes. For instance, a 2017 study by Zhang et al. discovered that DANCR promotes chondrogenic differentiation of human synovium-derived mesenchymal stem cells (hSMSCs) by regulating the miR-1305-Smad4 axis. The researchers found that DANCR knockdown inhibited hSMSC differentiation into chondrocytes, while DANCR overexpression yielded the opposite effect. Moreover, they demonstrated that DANCR regulates the expression of miR-1305, which in turn controls the expression of the transcription factor Smad4, a key regulator of chondrogenic differentiation [25]. Wang et al. published a study investigating the role of DANCR in the osteogenic differentiation of PDLSCs. They observed a significant decrease in DANCR expression during osteogenic differentiation of PDLSCs, and that the knockdown of DANCR promoted osteogenic differentiation as evidenced by increased expression of osteogenic markers. They concluded that DANCR serves as a negative regulator of osteogenic differentiation in PDLSCs [26].

Collectively, these studies suggest that DANCR plays a significant role in the regulation of differentiation processes across various cell types. Its tissue-specific expression and its capacity to act as a molecular sponge for microRNAs render it a compelling target for further research and potential therapeutic intervention. Furthermore, comprehending the mechanisms through which DANCR regulates differentiation may offer broader insights into the role of non-coding RNAs in cellular differentiation and development.

A study by Zhu and Xu established that DANCR regulates osteoblast differentiation by targeting the epigenetic regulator EZH2 and modulating the expression of Runx2, a pivotal transcription factor in osteoblast differentiation. The researchers found that the knockdown of DANCR inhibited osteoblast differentiation and diminished the expression of Runx2, while the overexpression of DANCR elicited the opposite effect. They posited that DANCR acts as a negative regulator of EZH2, thereby promoting osteoblast differentiation [27]. Similarly, a study by Jia et al. examined the role of DANCR in the osteogenic differentiation of PDLSCs. They found that the knockdown of DANCR enhanced the expression of osteogenic markers and boosted the osteogenic differentiation of PDLSCs, while DANCR overexpression had an inverse effect. They concluded that DANCR acts as a negative regulator of osteogenic differentiation in PDLSCs [28].

Human bone marrow mesenchymal stem cells (hBMSCs) were studied to discern the role DANCR plays in their growth and osteogenic differentiation. The researchers discovered that DANCR knockdown inhibited both the proliferation and osteogenic differentiation of hBMSCs, while overexpression of DANCR had the opposite impact. They proposed that DANCR fosters osteogenic differentiation by activating the p38 MAPK signaling pathway [29]. In sum, these studies collectively suggest that DANCR plays a critical role in regulating osteoblast and mesenchymal stem cell (MSC) differentiation. Its capability to control the expression of key transcription factors and signaling pathways makes it an attractive candidate for further exploration and possible therapeutic interventions. Additionally, understanding the molecular mechanisms by which DANCR governs osteoblast and MSC differentiation could have far-reaching implications for our comprehension of how non-coding RNAs contribute to bone development and homeostasis. Bone disorders such as osteoporosis and bone metastases are linked to DANCR dysregulation. Bone homeostasis and osteogenic differentiation are disturbed by aberrant expression. By specifically targeting DANCR, therapeutic potential is shown for the restoration of normal bone metabolism, the modulation of signaling pathways associated with bone, and the development of novel treatments for a variety of bone illnesses, such as osteoporosis and bone malignancies.

## 6 H19

lncRNA H19, located on human chromosome 11p15.5, serves as an essential regulator in osteogenesis and bone regeneration. It is involved in numerous biological processes, including but not limited to development, differentiation, and cellular growth. Recent research has unveiled H19’s pivotal role in osteoporosis and bone tissue regeneration. Elevated H19 levels have been observed in serum or plasma samples of osteoporotic patients. These findings imply a potential involvement of H19 in osteoporosis pathogenesis and bone metabolism regulation.

A study showed the upregulation of H19 in patients with osteoporosis [30]. It was observed that the overexpression of H19 could both encourage osteoclast formation and inhibit osteoblast differentiation. Conversely, the downregulation of H19 ameliorated osteoporosis by promoting osteoblast differentiation and restraining osteoclast formation. These findings lead to the inference that H19 serves as a prospective therapeutic target for the treatment of osteoporosis. Zhou et al. corroborated the role of H19 in regulating osteogenic differentiation and bone regeneration [31]. Their research indicated an upsurge in H19 expression during osteogenic differentiation in MSCs Knocking down H19 expression was found to hinder osteogenic differentiation and suppress bone regeneration [32].

Moreover, H19 advances the differentiation of stem cells from the apical papilla (SCAPs) into odontoblasts responsible for dentin formation. Li et al. identified this effect as mediated via the miR-141/SPAG9 pathway [33]. Experimental data, inclusive of histological examinations and OCN abundance comparisons, corroborated these observations. Behera et al. disclosed that exosomal H19 promotes both osteogenesis and angiogenesis in CBS-heterozygous mice [34]. These exosomes could be assimilated by osteoblasts and endothelial cells, thus bolstering their proliferation and migration. Mechanistically, H19 regulated angiopoietin-1/Tie2-NO signaling, culminating in the activation of osteogenesis and angiogenesis pathways.

**Figure 2:**
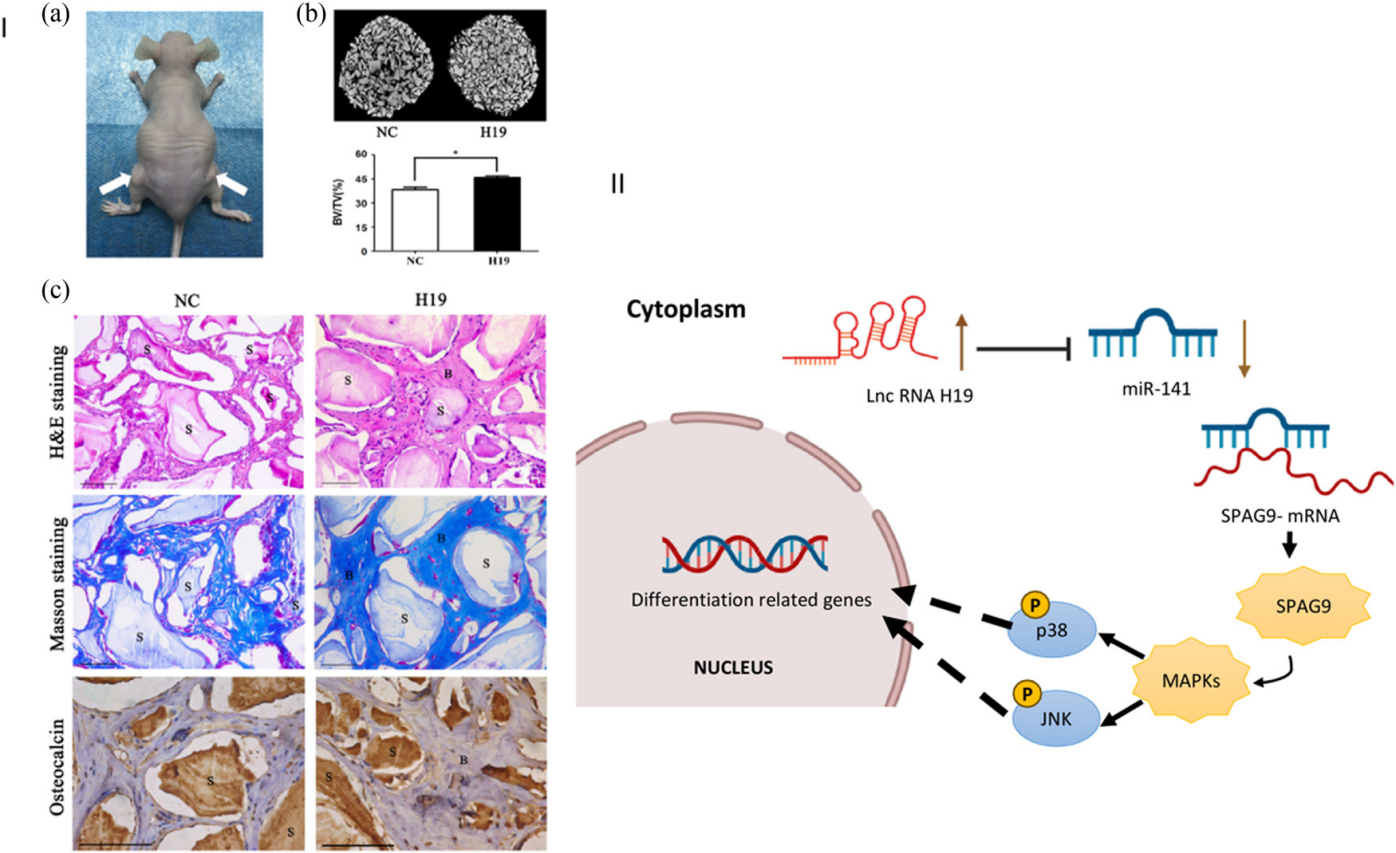
For the *in vivo* experiments, subcutaneous transplantation was performed in 5-week-old BALB/c homozygous nude mice for 8 weeks, with stem cells from the apical papilla (SCAPs) in NC and H19 groups. (a) Three-dimensional micro-CT imaging was used to reconstruct the tissue-engineered bone constructs, and the percentage of new bone volume to tissue volume (BV/TV) was calculated for each group. (b and c) The results showed significant differences in BV/TV between the NC and H19 groups, with higher values observed in the H19 group. Histological analysis was also performed, including H&E staining, Masson staining, and immunohistochemical staining of osteocalcin, which revealed the presence of bone/dentin-like tissues around the scaffold in both groups, with higher levels of osteocalcin staining in the H19 group. II. These findings suggest that H19 may enhance bone regeneration and have potential therapeutic applications in bone tissue engineering. Images obtained from Li et al. [33].

Diverging into the realm of oncology, H19 has been implicated in various cancer types and has been studied for its dual roles as a tumor suppressor and a cancer promoter [35,36,37,38]. Multiple studies have revealed its regulatory effect on various microRNAs and signaling pathways that could make it a potential therapeutic target for cancer treatment as well [39]. Therefore, while H19 offers promising avenues for therapeutic applications in osteoporosis and bone regeneration, its role remains complex and not fully delineated. Studies present a dichotomy where H19 both inhibits and promotes osteoblast differentiation, depending on the context. Chen et al. have even suggested that its dysregulation may be integral to the pathogenesis of osteoporosis [30]. H19 affects bone metabolism and osteoblast development in a number of ways. It regulates microRNA activity by acting as a competing endogenous RNA (ceRNA) [40], which affects the expression of osteogenic genes. H19 affects osteoblast development pathways through its interactions with transcription factors and chromatin modifiers. Knowing these pathways could reveal possible osteoporosis treatment targets. When treating osteoporosis, targeting H19 may have unintended consequences, such as interfering with regular cell functions that H19 regulates in other tissues. Furthermore, modifying H19 expression may have an impact on the ratio of osteoblast to osteoclast activity, which could have unanticipated consequences for the dynamics of bone remodeling and overall bone health. Overall, H19 emerges as a crucial regulatory molecule in osteogenesis and bone tissue regeneration. Its precise molecular mechanisms remain an area ripe for further investigation, with the ultimate objective of developing efficacious therapeutic strategies predicated on H19 regulation (Figure 2).

## 7 lncRNA for enhancing osteogenesis 3 (lncRNA-ES3) and rhabdomyosarcoma 2-associated transcript (RMST)

This lncRNA helps bone cells grow and mature. These transcripts control gene translation and cellular activities in many biological processes, including bone development. lncRNA-ES3 is one of several bone-developing lncRNAs. lncRNA-ES3, a 1,874-base-pair gene, was found in a genome-wide study of human mesenchymal stem cell osteogenic development. lncRNA-ES3 has been found to play a role in regulating the calcification and senescence of vascular smooth muscle cells (VSMCs) induced by high glucose levels [41]. The lncRNA interacts with miR-34c-5p to target the pro-apoptotic protein BMF, ultimately leading to reduced apoptosis and increased calcification and senescence of VSMCs. This study suggests that lncRNA-ES3 may serve as a potential therapeutic target for treating vascular calcification associated with diabetes. RMST affects bone and osteoblast formation. RMST is an lncRNA that plays a critical role in regulating BMP9-induced osteogenic differentiation of MSCs. The study by Zhang et al. found that RMST acts as an important mediator of BMP9-induced osteogenesis by antagonizing Notch-targeting microRNAs [42]. lncRNA-ES3 controls the development of osteoblasts via modifying the BMP and Wnt/β-catenin signaling pathways. Through the control of Sox9 and Runx2, essential transcription factors in chondrogenesis and osteogenesis, respectively, RMST affects the development of bones. Comprehending these pathways clarifies the mechanics behind lncRNA-mediated regulation of bone growth and equilibrium [43]. It may be possible to determine whether lncRNA-ES3, RMST, and recognized osteogenic regulators including miRNAs, transcription factors, and signaling molecules work in concert or against one another to create bones by investigating possible interactions between these entities. This thorough comprehension would disclose complex regulatory networks controlling osteogenesis and bone homeostasis. This implies that RMST might be a useful therapeutic target to encourage the growth and repair of new bone. Since they alter important regulatory pathways, targeting lncRNA-ES3 and RMST may lead to more targeted therapeutic approaches and better patient outcomes in the treatment of bone ailments.

## 8 Urothelial cancer associated 1 (UCA1)

UCA1 controls the growth and hardening of osteoblasts during bone remodeling. This lncRNA affects cell division, death, movement, and infiltration. Recently, UCA1 has been linked to bone development and remodeling, suggesting it could be a target for treating various bone disorders. UCA1, a 1.4-kb long lncRNA, was first identified as a cancer gene in bladder urothelial cancer. It is active in many organs and plays roles in various cell processes, new studies show. Among them, lncRNA UCA1 has a critical role in controlling the growth and change of osteoblasts, the transformation of hBMSCs into cartilage cells, and cell growth in bone cancer.

In a study by Zhang et al. UCA1 was found to influence osteoblast growth and change by regulating BMP-2 expression. BMP-2 is a key growth factor important for bone formation [44]. The researchers showed that reducing UCA1 lowered BMP-2 levels and held back osteoblast growth and change. On the other hand, increasing UCA1 boosted BMP-2 levels and encouraged osteoblast growth and change. Mechanistically, UCA1 controls BMP-2 levels by binding to and holding back miR-204-5p, a molecule that usually reduces BMP-2 levels. So, UCA1 helps bone formation by increasing BMP-2 levels. Likewise, a study by Shu et al. showed that UCA1 helps the cartilage-making process of hBMSCs by controlling certain pathways [45]. When UCA1 levels were raised, cartilage formation improved, and when they were lowered, it got worse. Through a number of methods, UCA1 controls the proliferation and differentiation of osteoblasts. By sponging miR-204-5p, it increases osteogenic differentiation and upregulates Runx2 expression. In addition, it stimulates the PI3K/Akt pathway, which increases osteoblast proliferation. These mechanistic findings highlight the critical function of UCA1 in bone homeostasis and development [45].

In a study by Li et al., UCA1 was found to boost cell growth in bone cancer by turning off the PTEN/AKT signaling pathway, which is important for controlling cell growth and survival [46]. Reducing UCA1 held back cell growth and caused cell death while increasing UCA1 boosted cell growth and prevented cell death. Mechanistically, UCA1 did this by interacting with HIF-1α, a molecule that controls the activity of many genes, including UCA1 itself. In summary, these studies show that UCA1 has a key role in bone formation and could be a target for new treatments. Clinical applications for treating bone diseases could benefit from targeting UCA1. Its potential as a therapeutic target is suggested by preclinical research indicating its regulatory involvement in osteoblast function. To achieve its therapeutic effects in human bone problems, clinical studies must be conducted after comprehensive confirmation of safety profiles, efficacy, and delivery strategy modification. Targeting UCA1 for bone disorders presents a number of challenges, including the necessity for effective delivery mechanisms, off-target effects brought on by its involvement in numerous cellular processes, and potential individual differences in expression levels. Before starting a therapy, it is also necessary to thoroughly evaluate the complex regulatory networks including UCA1 and long-term safety issues. More research is needed to fully understand how UCA1 works and to see if it can be used for treating bone diseases.

## 9 Taurine up-regulated gene 1 (TUG1)

TUG1, which influences bone cell development and mineralization, may affect osteoporosis and other bone diseases. This lncRNA has multiple roles, including bone development. Recent research has highlighted the function of lncRNAs like TUG1 in controlling MSC differentiation into osteoblasts. The 7.1-kilobase lncRNA TUG1 has been implicated in controlling cell growth, apoptosis, and differentiation across a wide range of cell types and tissues. In the context of bone formation, TUG1 has been found to promote osteogenic differentiation of human periodontal ligament stem cells (hPDLSCs) by sponging microRNA-222-3p (miR-222-3p) to negatively regulate the Smad2/7 signaling pathway. By interacting with miRNAs like miR-204-5p and releasing their repression on osteogenic transcription factors like Runx2, TUG1 controls osteogenic differentiation and bone production. Furthermore, TUG1 may influence Wnt/β-catenin and other signaling pathways. These revelations deepen our understanding of TUG1’s function in bone biology. Some studies looked into how TUG1 affects osteogenic development in hPDLSCs. During osteogenic differentiation of hPDLSCs [47,48], TUG1 expression was observed to be upregulated, and knockdown of TUG1 significantly suppressed osteogenic differentiation. Mechanistically, TUG1 was shown to act as a sponge for miR-222-3p, which led to the upregulation of Smad2/7, key regulators of the osteogenic differentiation pathway. Further experiments showed that overexpression of miR-222-3p could reverse the effect of TUG1 knockdown on osteogenic differentiation.

The impact of TUG1 reduction on osteoblast viability, migration, and differentiation was studied [47]. They found that TUG1 knockdown significantly reduced cell viability and migration, and inhibited osteoblast differentiation. Mechanistically, TUG1 was shown to act as a sponge for miR-214, which led to the downregulation of the osteogenic transcription factor Runx2. Further experiments showed that overexpression of miR-214 could reverse the effect of TUG1 knockdown on osteoblast differentiation. Hao et al. investigated the role of TUG1 in promoting osteoblast proliferation and differentiation by regulating the miR-545-3p/cannabinoid receptor 2 (CNR2) axis. They found that TUG1 expression was upregulated during osteogenic differentiation of hBMSCs, and knockdown of TUG1 significantly reduced osteogenic differentiation. Mechanistically, TUG1 was shown to act as a sponge for miR-545-3p, which led to the upregulation of CNR2 expression. Further experiments showed that overexpression of miR-545-3p could reverse the effect of TUG1 knockdown on osteogenic differentiation [49]. In a study by Özgür et al., the authors investigated the differential expression of lncRNAs during genotoxic stress-induced apoptosis in HeLa and MCF-7 cells. They found that TUG1 expression was upregulated during apoptosis in both cell lines, suggesting a potential role for TUG1 in regulating apoptosis [50]. While this study did not specifically investigate the role of TUG1 in bone formation, it highlights the potential importance of TUG1 in regulating cell survival and apoptosis, which are critical processes during bone formation.

The study by Teng et al. aimed to investigate the role of TUG1 in osteoporosis and its effect on the osteogenic differentiation of BMSCs. The researchers found that TUG1 was downregulated in osteoporotic patients, and overexpression of TUG1 promoted osteogenic differentiation of BMSCs [51]. This suggests that TUG1 might be a potential target for treating osteoporosis. Another study by Zhang et al. explored the role of TUG1 in inhibiting osteogenesis of BMSCs after irradiation. The researchers found that TUG1 inhibited the osteogenic differentiation of BMSCs by suppressing the expression of Smad5, a critical mediator of bone formation. This study suggests that TUG1 might be a potential target for preventing radiation-induced bone loss [52]. In a study by Liu et al., the researchers investigated the potential use of lncRNAs, including TUG1, as biomarkers for regulating the osteogenic differentiation process in bone defect management. The researchers found that TUG1 was significantly upregulated during the osteogenic differentiation of BMSCs and that its overexpression enhanced the osteogenic differentiation of BMSCs. This study suggests that TUG1 might be a potential biomarker for bone defect management [53].

One such study by He et al. investigated the role of TUG1 in the osteogenic differentiation of PDLSCs. The researchers found that TUG1 facilitated the osteogenic differentiation of PDLSCs by interacting with Lin28A, a critical regulator of stem cell differentiation. This study suggests that TUG1 might be a potential therapeutic target for promoting bone regeneration in periodontal diseases [54]. According to Liu et al. scientists looked into how TUG1 controls osteoblast proliferation and differentiation via the Wnt/-catenin signaling system. By activating the Wnt/-catenin signaling pathway, TUG1 was discovered to stimulate osteoblast proliferation and differentiation, as previously hypothesized. This study suggests that TUG1 might be a potential target for treating osteoporosis and other bone-related disorders by promoting bone formation [55].

Additionally, Wang et al. studied the role of TUG1 in regulating osteoblast proliferation and apoptosis under fluid shear stress. The researchers found that TUG1 regulated osteoblast proliferation and apoptosis via the TUG1/miR-34a/FGFR1 axis under fluid shear stress. This study suggests that TUG1 might be a potential target for promoting bone formation under mechanical loading conditions. Overall, these studies indicate that TUG1 plays a crucial role in bone formation and its regulation [56]. TUG1 might be a potential therapeutic target for promoting bone regeneration in periodontal diseases and treating osteoporosis and other bone-related disorders by promoting bone formation. Moreover, TUG1 might be a potential target for promoting bone formation under mechanical loading conditions. However, further studies are needed to explore the precise mechanisms by which TUG1 regulates bone formation and its potential use as a therapeutic target for treating bone-related disorders. Additionally, the development of novel strategies for delivering TUG1 to bone tissue would be necessary for its potential clinical use as a therapeutic target. The potential for TUG1-targeted treatments to be used in the treatment of bone diseases in a clinical setting appears intriguing. Transitioning from preclinical research to clinical trials necessitates meticulous validation of effectiveness, safety profiles, and refinement of administration techniques. These improvements show great potential in meeting the unfulfilled requirements of managing bone diseases. Targeting TUG1 for therapeutic intervention in bone diseases presents a number of challenges, including the need for effective delivery mechanisms, off-target effects resulting from its diverse activities, and individual variations in expression levels. The complex regulatory networks around TUG1 and long-term safety issues make a full review necessary before clinical deployment.

Overall, these studies suggest that TUG1 plays a crucial role in bone formation and its regulation. TUG1 might be a potential target for treating osteoporosis and preventing radiation-induced bone loss. Moreover, TUG1 might be a potential biomarker for bone defect management. However, further studies are needed to explore the precise mechanisms by which TUG1 regulates bone formation and its potential use as a therapeutic target or biomarker for bone-related disorders.

## 10 Nuclear enriched abundant transcript 1 (NEAT1)

NEAT1 controls osteoblast formation and mineralization, indicating it helps bone growth and maintenance. NEAT1 is increasingly important in gene translation and other biological functions. Nuclear lncRNA NEAT1 creates paraspeckles and is highly conserved across species. Recent studies have examined NEAT1’s role in osteogenesis and bone formation. NEAT1, an lncRNA, has been reported to play a crucial role in bone formation. Multiple studies have indicated the involvement of NEAT1 in the regulation of osteoblast function and bone regeneration. NEAT1 modulates osteoblastogenesis and bone mineralization through several pathways. It engages with miRNAs, such as miR-204-5p, to regulate important osteogenic transcription factors including Runx2. Furthermore, NEAT1 has the potential to affect signaling pathways such as Wnt/β-catenin, which in turn can have an impact on the differentiation of osteoblasts and the mineralization of bones. This enhances our understanding of NEAT1’s significance in the field of bone biology. In this article, we will review the latest findings related to the function of NEAT1 in bone formation.

One study by Zhang et al. explored the role of NEAT1 in hBMSCs and found that it promotes osteogenic differentiation by regulating the BMP1 signaling pathway. The study showed that NEAT1 positively regulates BMP1 expression by targeting miR-29b-3p, which is a negative regulator of BMP1. The results showed that the overexpression of NEAT1 could enhance the osteogenic differentiation of hBMSCs, as evidenced by the increased expression of osteogenic markers such as ALP, COL1A1, and RUNX2. The study also demonstrated that the knockdown of NEAT1 led to decreased osteogenic differentiation of hBMSCs [43].

**Figure 3:**
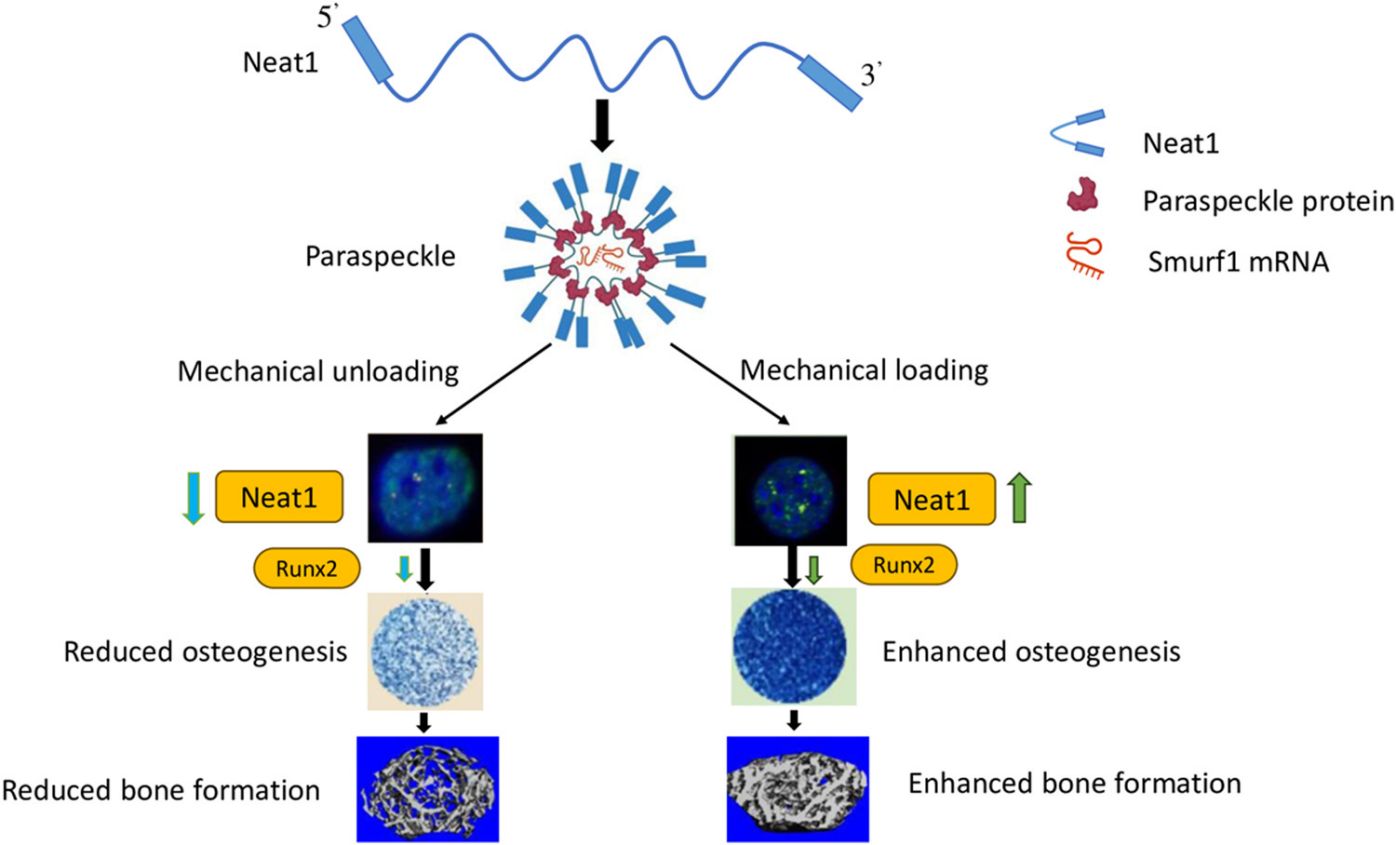
This proposed model suggests that Neat1-containing paraspeckles play a crucial role in the regulation of osteoblasts in response to mechanical stimulation. These paraspeckles act as mechanotransducers and help in the regulation of bone formation. Images obtained from Liu et al. [57].

In another study, Liu et al. investigated the mechanosensitive function of NEAT1 in osteoblasts. The study showed that NEAT1 is involved in the regulation of osteoblast function through the paraspeckle-dependent Smurf1 mRNA retention. The results showed that NEAT1 positively regulates the expression of Smurf1 by promoting its mRNA retention in paraspeckles. Smurf1 is a negative regulator of BMP signaling, and its upregulation can inhibit osteoblast differentiation. The study demonstrated that the knockdown of NEAT1 led to decreased osteoblast function, as evidenced by the reduced expression of osteogenic markers such as ALP, OCN, and RUNX2 [57]. The article "The mechanosensitive lncRNA Neat1 promotes osteoblast function through paraspeckle-dependent Smurf1 mRNA retention" explores the role of the lncRNA Neat1 in promoting osteoblast function. The study found that Neat1 is upregulated in response to mechanical stimulation and promotes osteoblast differentiation and mineralization through the regulation of Smurf1 mRNA stability. The authors suggest that Neat1 may be a potential therapeutic target for treating bone-related diseases such as osteoporosis [57]. This proposed model suggests that Neat1-containing paraspeckles play a crucial role in the regulation of osteoblasts in response to mechanical stimulation. These paraspeckles act as mechanotransducers and help in the regulation of bone formation (Figure 3). Chen et al. studied the role of exosomal NEAT1 in bone regeneration. The study showed that NEAT1 derived from endothelial cells could be transported to macrophages through exosomes, promoting their polarization towards an M2 phenotype. The study demonstrated that NEAT1 positively regulates the expression of DDX3X, which is involved in the NLRP3 inflammasome pathway, and promotes M2 macrophage polarization. The results showed that the overexpression of NEAT1 led to enhanced bone regeneration in a rat calvarial defect model [58].

Zhao et al. explored a novel ceRNA regulatory network involving NEAT1 in osteoblast autophagy and osteoporosis. The study demonstrated that NEAT1 acts as a sponge for miR-466f-3p and regulates the expression of its mRNA target, Atp6v0d2, which is involved in autophagy regulation. The study showed that the overexpression of NEAT1 could promote autophagy in osteoblasts and enhance osteoblast differentiation, as evidenced by the increased expression of osteogenic markers such as ALP, OCN, and RUNX2. The study also demonstrated that the knockdown of NEAT1 led to decreased bone mass and increased bone resorption in an ovariectomized mouse model of osteoporosis [59]. The lncRNA NEAT1 plays an important role in bone formation by regulating the lineage fate of bone marrow-derived mesenchymal stem cells (BMSCs). Therapies that specifically target NEAT1 have the potential to be used in the treatment of bone diseases. The transition from preclinical research to clinical trials requires thorough validation of both efficacy and safety profiles, as well as the optimization of delivery strategies. These improvements could provide new ways to treat diseases, highlighting the importance of NEAT1 in bone disorders and medical treatment. The challenges in targeting NEAT1 for therapeutic intervention in bone illnesses encompass off-target effects arising from its multifunctional activities, heterogeneity in expression levels between people, and the requirement for effective delivery mechanisms. A thorough assessment of safety profiles and refinement of therapeutic approaches are essential for the successful implementation of clinical translation. NEAT1 can impair the mitochondrial function and pluripotency maintenance of BMSCs during skeletal aging, leading to a reduction in their osteogenic differentiation potential. This study suggests that NEAT1 could be a potential target for therapeutic interventions to enhance bone formation and prevent age-related bone loss [60].

## 11 Conclusion

**Figure 4:**
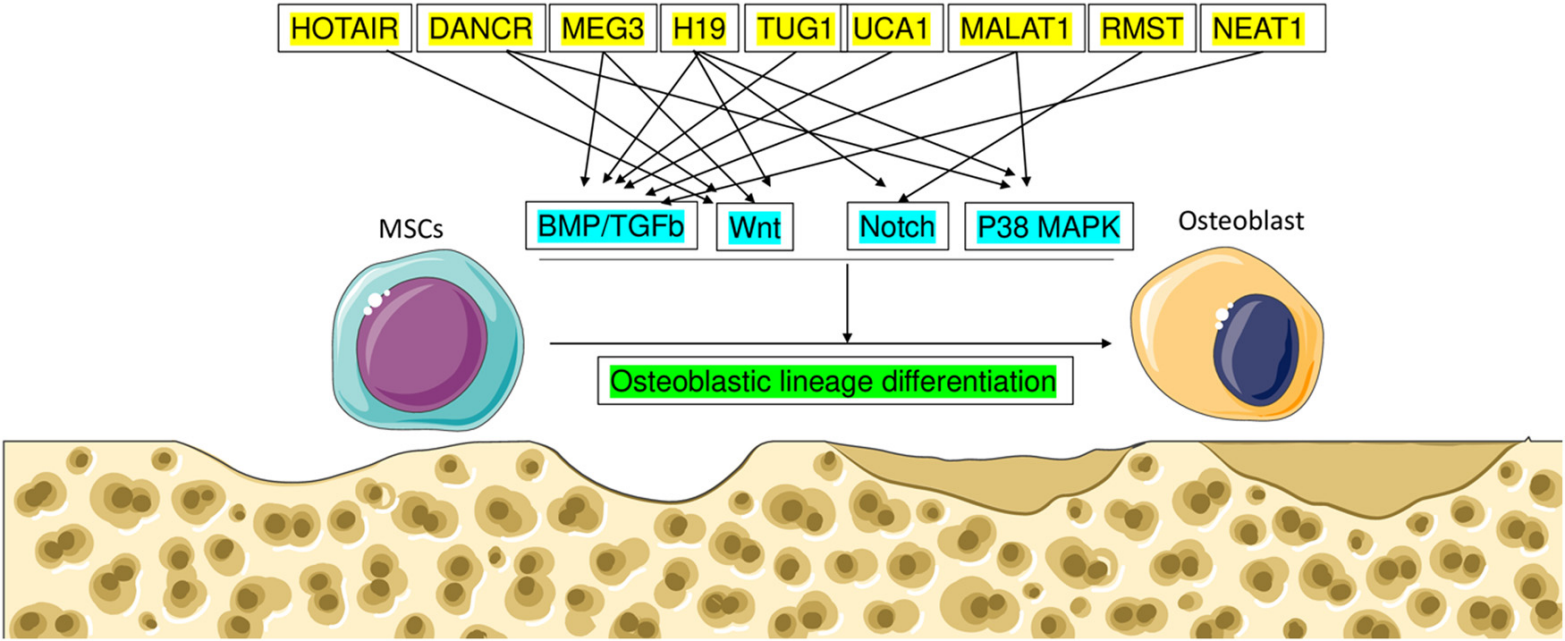
The osteogenic process is controlled by lncRNA through BMP, Wnt, Notch, and MAPK signaling.

In conclusion, the expanding field of lncRNAs in bone formation underscores their crucial roles in maintaining bone strength and health. The studies reviewed demonstrate the diverse ways in which lncRNAs contribute to bone growth, hardening, and maintenance. Notably, lncRNAs such as HOTAIR, MALAT1, DANCR, MEG3, H19, TUG1, UCA1, RMST, and NEAT1 have been extensively investigated for their involvement in bone regulation, acting through pathways like BMP, Wnt, Notch, and MAPK (Figure 4). These findings suggest the potential utility of lncRNAs in treating bone diseases like osteoporosis. However, further research is imperative to fully elucidate the mechanisms by which these molecules function and to develop targeted therapeutic interventions. The reviewed studies emphasize the crucial functions of lncRNAs in the process of bone formation and regulation. The authors clarify the processes by which lncRNAs are involved in the differentiation of osteoblasts, the process of bone remodeling, and the development of diseases. Comprehending these molecular interactions provides opportunities for novel therapeutic tactics and diagnostic methods in the field of bone biology, emphasizing the importance of lncRNAs as essential regulators in maintaining skeletal balance. Continued exploration of lncRNAs in bone development holds promise for improving care for individuals with bone diseases. Novel treatments for conditions such as osteoporosis may emerge from this research endeavor. Future studies could identify additional lncRNAs involved in bone growth and elucidate their mechanisms of action. Furthermore, understanding how lncRNAs interact with other pathways and factors to regulate bone cell growth and hardening is essential for developing targeted therapies to enhance bone growth and prevent bone loss. Moreover, lncRNAs may serve as diagnostic markers for early detection and improved treatment of bone diseases. Overall, advancing research in lncRNAs and bone growth holds significant potential for substantial improvements in the treatment of bone disorders and ultimately enhancing bone health and quality of life. Potential future investigations on lncRNAs in bone formation and disease could examine their functions in the differentiation of skeletal cells, the process of bone remodeling, and the pathogenesis of diseases related to the skeletal system. Exploring the relationships between lncRNA molecules and epigenetic regulators and signaling pathways has the potential to provide new targets for therapy and diagnostic biomarkers, which can contribute to the development of customized treatments for bone-related illnesses.
